# The Role of Dendritic Cell Subsets and Innate Immunity in the Pathogenesis of Type 1 Diabetes and Other Autoimmune Diseases

**DOI:** 10.3389/fimmu.2015.00288

**Published:** 2015-06-15

**Authors:** Jeffrey D. Price, Kristin V. Tarbell

**Affiliations:** ^1^Diabetes, Endocrinology, and Obesity Branch, Immune Tolerance Section, National Institute of Diabetes and Digestive and Kidney Diseases, National Institutes of Health, Bethesda, MD, USA

**Keywords:** dendritic cells, autoimmunity, type 1 diabetes, innate immunity, T cell tolerance, antigen presentation

## Abstract

Dendritic cells (DCs) are key antigen-presenting cells that have an important role in autoimmune pathogenesis. DCs control both steady-state T cell tolerance and activation of pathogenic responses. The balance between these two outcomes depends on several factors, including genetic susceptibility, environmental signals that stimulate varied innate responses, and which DC subset is presenting antigen. Although the specific DC phenotype can diverge depending on the tissue location and context, there are four main subsets identified in both mouse and human: conventional cDC1 and cDC2, plasmacytoid DCs, and monocyte-derived DCs. In this review, we will discuss the role of these subsets in autoimmune pathogenesis and regulation, as well as the genetic and environmental signals that influence their function. Specific topics to be addressed include impact of susceptibility loci on DC subsets, alterations in DC subset development, the role of infection- and host-derived innate inflammatory signals, and the role of the intestinal microbiota on DC phenotype. The effects of these various signals on disease progression and the relative effects of DC subset composition and maturation level of DCs will be examined. These areas will be explored using examples from several autoimmune diseases but will focus mainly on type 1 diabetes.

## Introduction

Dendritic cells (DCs) play a vital role in host immunity by inducing innate inflammatory responses to pathogens, efficiently priming naïve T cells, activating memory T cells, and promoting B cell activation. However, DCs are also integral in maintaining steady-state immune homeostasis by continually presenting tissue-derived self-antigens to CD4^+^ and CD8^+^ T cells in the absence of inflammatory signals, leading to tolerance against those self-antigens. DCs can affect induction of both immunity and tolerance in several ways, including at the level of DC development, the relative composition of DC subsets, and the extent of DC maturation.

Autoimmune diseases occur when autoreactive T and B cells escape negative selection in the thymus and bone marrow, respectively, followed by breaks in peripheral tolerance mechanisms that disrupt immune system homeostasis. Antigen-presenting cells (APCs), including DCs, play a central role both in the initial thymic selection of the T cell repertoire and in maintaining peripheral T cell tolerance for autoreactive cells. DCs affect autoimmune diseases including T cell-centric diseases such as type 1 diabetes (T1D) and multiple sclerosis (MS) and diseases thought to be mediated by B cells and antibodies, such as systemic lupus erythematosus (SLE). For example, in non-obese diabetic (NOD) mice, a model for T1D, DCs can modify pathogenesis by appearing in pancreatic islets early in life and presenting self-peptides to autoreactive T cells in the pancreatic lymph node (1). Thus, altering DC number or phenotype can affect disease progression (2).

**Figure 1:**
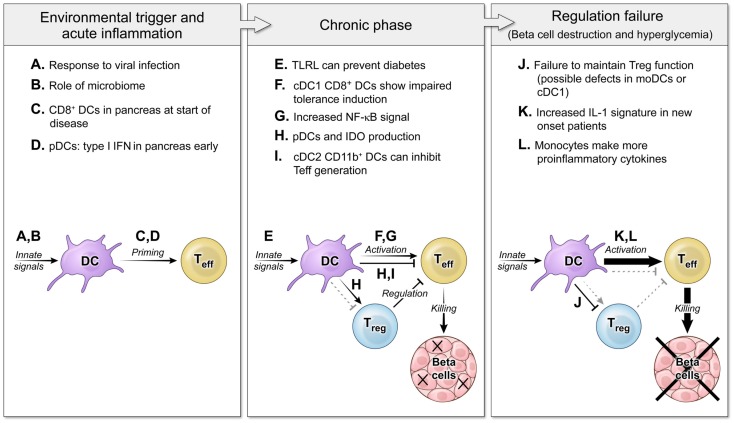
**The roles of DCs in autoimmune diabetes pathogenesis at several disease stages**. Three main phases of autoimmune pathogenesis occur in type 1 diabetes (with parallel stages found in other autoimmune diseases). Although defined by changes in T cell responses, these stages are controlled by DCs and innate immunity. First, an innate environmental trigger contributes to loss of peripheral tolerance and priming of autoreactive T cells (8–11). These innate signals can be infectious **(A)** or endogenous **(B)**, and the result is to activate DC populations that stimulate self-specific T cells **(C,D)**. Next, in the chronic phase of the disease, autoimmunity is tenuously balanced with regulation (12, 13). DCs continue to respond to innate stimuli, but now some TLRL block disease **(E)**. DCs interact with effector T cells and regulatory T cells to mediate both activating **(F,G)** and regulating interactions **(H,I)**. Finally, the balance tips to a failure of tolerance and tissue destruction mediated by non-productive interactions between DCs and Tregs **(J)** and DCs giving increased activating signals to Teff **(K,L)** (14, 15).

Early interplay between innate immunity and target tissues is often a hallmark of autoimmune disease (3, 4). The question of DC effects on autoimmune disease centers on the possibility that DCs could either induce or suppress autoreactive T cell responses and focuses on DC proteins that would affect those interactions. Initial studies of DC ablation showed that decreased DCs-induced autoimmunity because of an inability to maintain Tregs (5), yet other studies have demonstrated that a loss of DCs actually decreased disease severity by blocking activation of pathogenic cells (6, 7). In both autoimmune patients and murine models of autoimmunity, DCs exhibit alterations in phenotype or function that could be due to underlying genetic defects or the chronic inflammatory environment, and can affect both the initiation of disease and later failure of tolerance mechanisms that lead to tissue destruction such as loss of insulin-producing beta cells in T1D (Figure 1). Understanding the balance between the regulatory and pathogenic role of DCs is important for learning how to block autoimmunity in the clinic.

**Figure 2:**
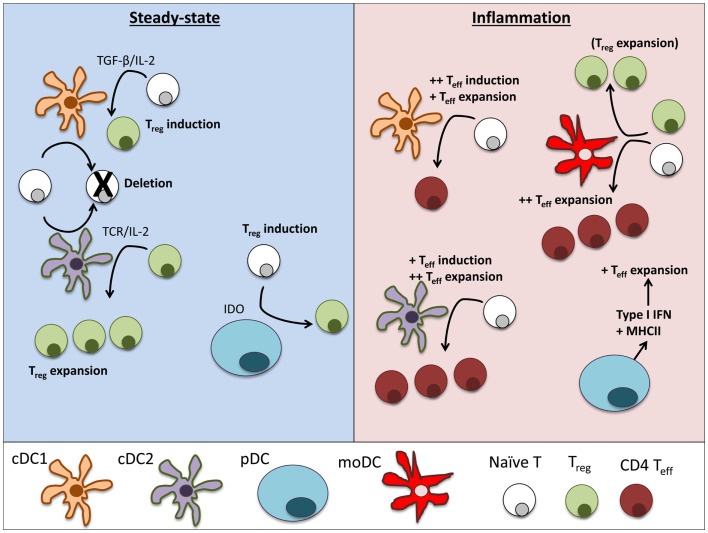
**Dendritic cell subsets perform particular functions in steady-state and inflammation**. In steady-state tissues (left panel), lymphoid-resident cDC1 and cDC2 bearing self-antigen can both suboptimally stimulate naïve CD4^+^ and CD8^+^ T cells and cause deletional tolerance of autoreactive cells. A subset of naïve CD4^+^ T cells that are stimulated by cDC1 will encounter TGF-β on the DC and induce Foxp3 and become a Treg. If a Treg is stimulated by cDC2, it will clonally expand that population of Tregs. pDCs have limited capacity to stimulate CD4^+^ T cells directly due to low MHCII and costimulatory molecule levels. Under certain conditions, pDCs have been demonstrated to produce IDO and induce Treg generation. During inflammation (right panel), cDCs mature and can stimulate effector T cell responses, including Th1 and Th17 cells often associated with autoimmune disease. cDC1 can induce strong Th1 responses from naïve cells and cDC2 are more efficient in expanding CD4 Teff. pDCs respond to inflammation by secreting large amounts of type I interferons that can significantly alter the pathogenesis of autoimmune diseases. Inflamed pDCs also upregulate MHCII, allowing efficient antigen presentation. moDCs mature from circulating monocytes (Ly6^+^ in mice, CD14^+^ in humans) as they enter inflamed tissues. moDCs are adept at inducing Th1 responses via secretion of IL-12, but can also expand Tregs in some circumstances.

The lack of uniform nomenclature and definitions of particular DC populations can make comparison of data from different groups difficult. Therefore, it is helpful to use the recent simplified nomenclature of cDC1, cDC2, pDC, and monocyte-derived cells (Figure 2) that is based primarily on ontogeny with further specialization depending on location, and highlights the match between mouse and human DC subsets (16). Under this system, steady-state DCs can be broken down into three main subsets based on developmental origin, surface markers, and function: plasmacytoid DCs (pDCs) that can produce high levels of type 1 interferons (IFN), and two conventional DC (cDC) subsets, cross-presenting cDC1 that expresses CD8 or CD103 in mice and cDC2 that express CD11b^+^ and efficiently stimulate CD4^+^ T cell proliferation (Table 1). In humans, cDC1 expresses BDCA3 and cDC2 express BDCA1 (17, 18). pDCs express BST2 and Siglec-H in mice and BDCA2 in humans (19, 20). Further specialization of these DC subsets occurs in peripheral tissues. Separate from these steady-state DC subsets, monocytes that are activated to express MHC II share some functional features with cDCs, such as expression of CD11b, but their overall gene expression patterns are much closer to monocytes than any DC subset (21, 22). Regardless, much of the literature refers to these monocyte-derived cells as DCs. Because GM-CSF can induce development of these monocyte-derived DCs *in vitro* from both human and mouse monocytes, this is a popular model, but it is important to recognize that they are a separate entity from cDCs. This review describes recent advances in our knowledge of the differential roles of particular DC subsets and activated monocytes for tolerance induction.

**Table 1:** **Parameters of DC subsets relating to autoimmunity**.

Dendritic cell subsets	Plasmacytoid DCs	cDC1	cDC2	Monocyte-derived DCs
Markers in mice	SiglecH, CD11c^−^ intermediate, B220^+^BST2^+^	DEC205, XCR1, Clec9A, CD8, or CD103	CD11b, DCIR2 (33D1)	CD11b CD11c MHCII high, DC-SIGN (CD209) DCIR2 negative
Markers in human	CLEC4C (BDCA2), CD123, CD11c low/neg	CD141 (BDCA3), XCR1, Clec9A,	CD1c (BDCA1)	CD14, MR (CD206)
Transcription factor	E2-2, Spi-B	Batf3, Irf8	IRF4, Notch2	Remains unclear
Precursor	CDP	CDP	CDP	cMoP
Main location	Bone marrow and peripheral lymphoid tissues	Lymph nodes and peripheral tissues	Spleen and peripheral tissues	Rare in steady state, inflammation recruits precursors from BM to lymphoid and peripheral tissues
Role in autoimmune pathogenesis	Needed for early type I IFN that elicits initiation of autoimmune diabetes	Efficient activation of CD8^+^ T cells through cross-presentation	Efficient proliferation of pathogenic CD4^+^ T cells	May expand effector T cells
Role in tolerance induction	Production of IDO, induction of IL-10 and Trl	Uptake of apoptotic cells and induction of new Tregs	Efficient proliferation of Tregs, induction of Th2?	*In vitro*-derived GM-CSF BM DCs expand Tregs and inhibit diabetes
Alteration in autoimmunity	More type 1 IFN production. In NOD, higher CD11c expression	Inability to induce CD4^+^ T cell tolerance and Treg induction	May be pathogenic and tolerogenic, but not clear due to lack of separation with monocyte-derived CD11b^+^ cells	In mice, more MHCIIhi monocytes due to inflammation. In T1D patients, monocytes make more pro-inflammatory cytokines
Reference	(4, 8, 9, 23–25)	(12, 26–28)	(29, 30)	(31–33)

## The Role of Specific DC Subsets in Autoimmunity

### Conventional DCs: CD8^+^ cDC1 and CD11b^+^ DCIR2^+^ cDC2

The different roles of specific DC subsets in eliciting autoimmune pathogenic responses versus tolerance induction are likely to be important for successful immunotherapy. cDC1s and cDC2s are primarily located in distinct anatomical locations in lymphoid tissues and process and present antigen on MHCI and MHCII differently, and thus cause different stimulation of CD8^+^ and CD4^+^ T cells (29). Ultimately, cDC1s efficiently cross-present antigens to CD8^+^ T cells, while cDC2s more efficiently stimulate CD4^+^ T cells, although either DC subset can stimulate both T cell subsets (26, 29). Using antigen-encoding chimeric antibodies that bind lectins differentially expressed by DC subsets to deliver antigen *in vivo* to specific DC subsets is one valuable tool that has made it possible to compare the T cell responses elicited *in vivo* by particular DC subsets. In mice, anti-DEC-205 antibodies have been used to efficiently target antigen to lymphoid-resident CD8^+^ DCs and migratory CD103^+^ cDC1s (34, 35). In non-autoimmune-prone mice, chimeric anti-DEC-205 antibodies elicit tolerance induction in both CD4^+^ and CD8^+^ T cells if no other inflammatory signals are added (i.e., steady-state tolerance), but can induce strong antigen-specific immunity if given with toll-like receptor (TLR) ligands and anti-CD40 (34, 36). Although less-studied, anti-DCIR2 has likewise been utilized to demonstrate that cDC2s are also tolerogenic *in vivo* for both T and B cell responses under steady-state conditions (29, 37).

In autoimmune-prone NOD mice, DEC-205^+^ cDC1s are able to induce tolerance in autoreactive CD8^+^ T cells (27) but antigen presented by these DCs stimulate Th1 responses in autoreactive CD4^+^ T cells even without exogenous maturation signals. This defect in steady-state tolerance is corrected by inhibition of CD40/CD40L interactions (12). Indeed, NOD CD8^+^ cDC1s express higher CD40 compared to C57Bl/6 CD8^+^ cDC1. By contrast, targeting antigen to DCIR2^+^CD11b^+^ cDC2s induce tolerogenic responses even in this chronic autoimmune environment and stimulation of T cells by DCIR2^+^ cDC2s can inhibit diabetes development (38).

Other studies have also suggested a regulatory role of CD11b^+^ cells in NOD mice, but it is not clear exactly what APC subsets are involved. Although tolerogenic CD11b^+^CD11c^+^ cells abrogate diabetes when directed to the pancreas via increased CCL2 (30), other work shows that CD11b^+^ DCs may be responsible for aberrant stimulation of beta-cell specific CD4^+^ T cells in NOD mice (23). Cells that are CD11b^+^CD11c^+^ include cDC2s and monocyte-derived cells. Some of the monocyte-derived cells express high levels of MHC class II, especially in inflammatory settings (22). However, DCIR2 [and the corresponding antibody clone 33D1 (39)] is clearly specific for cDC2 cells, and use of this marker can separate monocyte-derived cells from cDC2s.

In addition to effects on the pathogenic T cells, cDCs can induce and expand autoantigen-specific Tregs that can block or reverse autoimmune pathology (40). Because DC interactions with Tregs can be enhanced by expression of costimulators, such as CD86 (41, 42), activation or maturation of DCs can sometimes have paradoxical effects on autoimmunity and the optimal DC state for tolerance maintenance may be semi-mature. DC subsets have different effects on Tregs. cDC1 DEC-205^+^ DCs can induce FoxP3 perhaps via TGF-β expression, producing a regulatory compartment of autoreactive T cells (28). cDC2 CD11b^+^ DCs do not express TGF-β or efficiently induce Treg differentiation, but they expand existing populations of Tregs and can thus contribute to Treg-mediated suppression of autoimmunity (28, 43). However, factors other than TGF-β likely have a role in Treg induction by these DC subsets, as TGF-β may only have a minor role in conversion of Foxp3^−^ Treg precursors into Tregs (44). A recent study has shown the importance of migratory DCs in driving Treg responses (35). As these migratory DCs, which can express DEC-205, traffic from distal tissue sites containing antigens potentially relevant for autoimmune responses, defects in the ability of migratory DCs to induce or expand Tregs may inhibit self-tolerance and increase autoimmunity.

Like T1D, MS, and the associated mouse model, experimental autoimmune encephalomyelitis (EAE) are associated with aberrant T cell activation. Although DCs are not necessary for induction of EAE, several studies have demonstrated that DCs can permit immune invasion into the central nervous system (CNS) (45, 46). Both CD8^+^ and CD11b^+^ DCs are found in the CNS during EAE, and can play a role in activating pathogenic T cells (47). Ablation of DCs in EAE models do not alter disease incidence but exhibit amelioration of disease severity, indicating that DCs play a role in priming pathogenic T cells or generating the inflammatory milieu (48). CD11b^+^ DCs are located at the blood–brain barrier, and have been surmised to drive Th17 development that promotes EAE. Indeed, DCs are probably a major producer of inflammatory cytokines in the CNS during EAE induction and the acquisition of neuroantigens by DCs, as opposed to resident microglia, coincides with EAE symptom onset (49, 50).

Experimental autoimmune encephalomyelitis, and other induced autoimmune models, require immunization to break self-tolerance and initiate disease pathogenesis, and are models primarily for the effector phase of diseases such as MS and rheumatoid arthritis (51). But, immunization with a strong innate signal such as CFA may not be a good context to measure the state of innate immune and antigen-presenting function in a more chronic, but less dramatic, inflammatory environment such as that found during natural autoimmune pathogenesis. Therefore, in models such as EAE one can either intervene prior to immunization, when the mouse has no inflammation, or after immunization, when the inflammatory state may be distinct from the chronic inflammation that is found in tissue-specific autoimmune diseases. For example, several studies have shown that targeting self-antigen to DEC-205^+^ DCs to mice prior to inducing EAE can block disease, but the DCs are presenting antigen in a non-inflammatory context (35, 52, 53). Targeting antigen to DEC-205^+^ cDC1s in NOD mice after initiation of autoimmunity induces an effector response, and cDC1s express higher levels of CD40 in NOD mice even without addition of TLRL or other innate stimulus (12). It is probable that other innate and regulatory pathways are deregulated in the context of chronic autoimmunity; therefore, it is important that tests of antigen-specific tolerance induction are performed in models such as the NOD mouse that may better reflect the immune state of autoimmune patients.

An unusual cDC population termed as merocytic DCs has been identified that are CD11c^+^ MHCIIhi, but negative for both CD8 and CD11b (54). Interestingly, this population can acquire antigen from apoptotic fragments and is more numerous in NOD mice, a trait that maps to the insulin-dependent diabetes (*Idd)*13 genetic susceptibility locus (55). Transfer of purified merocytic DCs pulsed with beta cell antigen was shown to accelerate diabetes in NOD mice (56). However, a specific positive marker has not been identified for this population, making it difficult to ensure it is a uniform population and to determine if these cells more closely resemble cDC1 or cDC2.

### Plasmacytoid dendritic cells

Plasmacytoid DCs are significant contributors of type 1 IFN after stimulation via TLR7 or TLR9 and as such can facilitate autoimmunity as is clearly the case with SLE (8, 57). However, they can also play a significant role in regulation of immune responses by secreting IDO, inducing Tregs or inhibiting pathogenic responses (23, 24, 58, 59). Although evidence suggests that IFN-α from pDCs in the pancreas of NOD mice early (2–4 weeks of age) is important for initiation of autoimmune diabetes, other groups show that pDCs are only present in the islets later in the disease and play a protective role via IDO (4, 9, 23, 25). A recent human study shows increased IFN-α from peripheral blood pDCs in patients with type 1 diabetes (60). It is possible that pDCs may be playing both a pathogenic and a regulatory role in T1D, depending on disease stage and microenvironment, but this needs further study. pDCs can also be relevant in some contexts for antigen presentation. For example, depleting pDCs exacerbates EAE and MHCII expression is needed on pDCs to inhibit EAE (58, 59). Targeting of autoantigen to pDCs with antibodies against SiglecH (a lectin expressed specifically on pDCs) reduced CNS autoimmunity (61). Interestingly, although targeting antigen to pDCs via SiglecH-induced T cell regulation, antigen targeted to pDCs via BST2 resulted in immunity (62). Two possible explanations for the divergent response are that (1) siglecH has an ITIM motif, and when antibody binds, pDCs produce less IFN and (2) although BST2 is specific to pDCs in non-inflamed environments, many other APCs can upregulate BST2 with inflammation (63).

### Monocyte-derived cells

There are also several monocyte-derived cell populations that appear to have therapeutic potential and the ability to drive T cell tolerance via cell-intrinsic mechanisms or Treg induction. As discussed above, some studies identifying roles for CD11b^+^ cells may actually be studying a monocyte-related population, not cDCs. Bone marrow DCs generated *in vitro* with GM-CSF are monocyte derived. GM-CSF BMDCs efficiently stimulate proliferation of self-specific Tregs that can effectively block and reverse diabetes pathogenesis (31, 64). Both GM-CSF/IL-4-derived DCs and IL-10-derived DCs are tolerogenic monocyte-derived populations that can alter Treg populations and inhibit autoreactivity (65, 66). These tolerogenic DCs are being actively studied for possible therapies in human autoimmunity and for blocking medical complications such as graft-versus-host disease (67, 68). On the other hand, T1D patients have activated monocytes in the peripheral blood that make elevated levels of pro-inflammatory cytokines (32). Therefore, monocyte-derived DCs, like other DC subsets, also can provide both tolerogenic and immune activating signals that alter autoimmune pathogenesis (Figure 1).

## The Role of DC Development in Autoimmunity

Induction and maintenance of T cell tolerance can be affected by alterations in DC development, which occur via hematopoetic bone marrow precursors that become more specialized toward the DC lineage, starting with committed myeloid precursors (c-kit^+^CX3CR1^+^Lin^−^), monocyte and DC precursors (c-kit^+^CX3CR1^−^ Lin^−^), and committed DC precursors (c-kit^lo^CX3CR1^+^CD115^+^Lin^−^) (69). pDCs are an independent lineage that branch off from the cDCs at the CDP stage. TNF-α/iNOS-producing DCs and Gr-1^+^ inflammatory DCs differentiate from monocytes during inflammation via GM-CSF, but are rare in steady-state mice (14, 70, 71).

Flt3L is known to be essential for the development of DCs. pDCs and cDCs can be generated *in vitro* by culturing bone marrow cells (that include DC precursors) with Flt3L. Exogenous Flt3L boosts mouse and human DC numbers *in vivo*, including both cDC1 and cDC2 (72, 73). Increased Flt3L expression results in an increase in Tregs that correlates with the total number of CD11c^+^ cells (5). Studies using different timing of Flt3L treatment demonstrated that DCs can either ameliorate type 1 diabetes when given early in life or increase disease severity if Flt3L is given at later time-points when pre-existing autoimmune T cells are present (74). This underscores the dual nature of DC actions on T cells in autoimmune disease.

Dendritic cells in NOD mice exhibit alteration from development of DC subsets. cDC1 is underrepresented in NOD mice (75). Indeed, studies using Flt3L treatment of NOD mice increased CD8^+^ DC numbers and ameliorated disease, indicating that DCs are important for inhibiting diabetes progression (76). However, more recent studies have determined that Batf3-dependent DCs are necessary for induction of diabetes (77). Batf3 is a transcription factor that regulates cDC1 differentiation (78). These results highlight the dual roles that DCs play in autoimmune responses (77). NOD mice also express lower levels of IL-2 at steady-state than non-autoimmune-prone mice (79). IL-2 is a diabetes-associated gene located within the *Idd*3 loci. Although research has focused on the role of IL-2 on T cells and specifically Treg numbers in diabetes, IL-2 also affects DC development. The lower levels of IL-2 present in NOD mice lead to increased numbers of DCs and could play a role in aberrant T cell activation. The increased spleen pDC numbers observed in NOD but not *Idd3/5* mice, inversely correlate with respective IL-2 levels (80). Therefore, alterations in DC development can alter pathogenesis of diabetes and other autoimmune diseases.

## Genetic Susceptibility Genes can Alter Autoimmune Pathogenesis via Effects on Antigen Presentation and Innate Immunity

The genetic region with the highest risk association in most autoimmune diseases is MHCII (81), which links autoimmunity to antigen presentation. Exactly how particular alleles of MHCII confer susceptibility or resistance is still not clear after decades of extensive study, but the most widely held theory is that particular alleles direct repertoire selection in the thymus, and may affect the specificity of peripheral tolerance as well. Other genes associated with APCs have also been linked to various autoimmune diseases. For example, as stated previously in the section on pDCs, genes related to type 1 IFN are implicated in a number of autoimmune diseases (82). In models of diseases such as SLE, several studies have indicated that a loss of negative regulators of inflammation (such as A20, Shp1, and Blimp1) specifically in DCs lead to autoimmune phenotypes (83–86).

Some susceptibility genes, such as IL-2, protein-tyrosine phosphatase, non-receptor type 22 (PTPN22), and B lymphocyte-induced maturation protein 1 (BLIMP1), studied primarily for functional roles in lymphocytes also affect DC phenotype (83–87). BLIMP1 is a transcriptional repressor that modulates the MHCII loci, but can be ubiquitinated by the ubiquitin ligase Hrd1 to increase MHCII expression in DCs, and loss of Hrd1 protected against EAE (88). PTPN22 is a tyrosine phosphatase that is directly involved in TLR-mediated signaling and can profoundly affect type 1 IFN production in innate immune cells (89). In type 1 diabetes, many of the identified susceptibility loci affect APCs. Genes in the *Idd*4 locus can alter both IL-12 and IFN responses that affect antigen presentation and the type of T cell responses that ensue (90). Bone marrow chimera studies have shown that *Idd*3/5 and *Idd*9 can affect diabetes pathogenesis in T cell-independent, DC-dependent manner, although the particular DC subsets involved have not been elucidated (91, 92). *Idd3* encodes IL-21 in addition to IL-2, and polymorphic variants of IL-21 and its receptor have been implicated in genetic susceptibility to T1D (93). IL-21R-deficient DCs fail to acquire expression of CCR7 to shuttle between the pancreas and draining lymph nodes or efficiently express MHCII to induce autoreactive T cell pathogenesis (94). Data utilizing NOD mice congenic for the diabetes-resistant alleles at the *Idd3* and *Idd5* loci demonstrated that expression of these alleles is important in DCs for CD4^+^ T and CD8^+^ T cell tolerance. Therefore, the disease-altering effects of these two loci come from both lymphoid and non-lymphoid cells, and this may be a common feature of genetic risk.

## The Level of DC Maturation and Response to Environmental Stimuli can Alter Autoimmune Pathogenesis

Dendritic cells respond to numerous maturation signals during host responses. An array of innate receptors, including TLRs NOD-like receptors, RIG-I-like receptors, and AIM2-like receptors, as well as receptors for inflammatory cytokines, are expressed by DCs and act as environmental sensors. These receptors bind ligands from the local microenvironment and induce maturation of DCs, acting as a switch to induce effective adaptive immune responses. In addition to pathogen-associated molecular patterns, these innate receptors can bind endogenous ligands or danger-associated molecular patterns, which can alter DC phenotype and function. Host-generated chronic inflammation during autoimmune pathogenesis is one source of this non-pathogen-associated innate immune signal (3). Because of these activating signals, DCs in autoimmune individuals have an altered ability to induce tolerance [reviewed in Ref. (95)]. DCs play a major role in the homeostasis of regulatory T cells (Tregs) and can thus also indirectly influence effector T cell activation and tolerance via these cells. These interactions change at different stages of disease (Figure 1). Therefore, DCs can contribute both to the pathogenesis and regulation of autoimmunity.

The cytokine milieu in an autoimmune-prone individual or animal can have important effects on DC phenotypes. Alterations in DCs in NOD mice have been linked to diabetes progression, and a type 1 interferon signature has been noted prior to T cell activation within the pancreas (96, 97). GM-CSF BMDCs from NOD mice express increased NF-κB and higher levels of the Th1-inducing cytokine IL-12 and adenosine deaminase, although it is not yet clear if cDCs display similar responses (33, 98, 99). Migratory DC populations express lower levels of the immunomodulatory cytokine IL-10 in NOD mice as compared to non-autoimmune-prone strains (13). TNF-α, which can be produced in response to environmental stimuli that signal via pattern recognition receptors, may be one major modulator of DC function in autoimmune disease. Mice treated neonatally with TNF-α exhibited more rapid diabetes progression that correlated with increased expression of costimulatory proteins on CD11b^+^ DC subsets, while decreasing the number of CD8^+^ DC in the pancreatic lymph nodes (100).

Pro-inflammatory DC function has been observed in MS, and interferon-β is used to treat the disease. Although the precise mechanism is not clear, IFN-β may act on DCs by inhibiting trafficking or reducing T cell activation, possibly via DC apoptosis (101, 102). By contrast, type 1 interferons have been prominently implicated in SLE pathogenesis, linking the disease to pDCs. Immune cells from SLE patients display a type 1 IFN signature, and their pDCs have a greater capacity for stimulating pathogenic T cells than pDCs from control patients (103). In new-onset T1D patients, an IL-1 signature has been measured, yet mouse models of T1D indicate an early role for type 1 IFN and T1D patients may have increases in IFN-α-producing pDCs (60, 104). Therefore, the nature of the inciting innate responses is not the same for all autoimmune diseases, and more work needs to be done to define the innate landscape in both human autoimmunity and the corresponding mouse models.

In addition to cytokines, surface costimulatory markers are altered in DCs from NOD mice. Our work has demonstrated that CD8^+^ DCs from NOD mice express increased levels of CD40 and that this increase alters their stimulatory capacity for self-specific CD4^+^ T cells (12). In the context of autoimmune gastritis, migratory DCs from the stomach also express a semi-activated phenotype with small increases in CD40, CD86, and MHCII. However, unlike the shift to effector T cell responses in NOD mice, these semi-activated DCs are refractory to TLR stimulation and maintain their ability to induce tolerant CD4^+^ T cells even in the presence of DC maturation factors (105). NOD mice that lack the interaction between CD28 on T cells and CD80/CD86 on APCs display accelerated disease and lack Tregs, showing that these positive costimulators associated with inflammation are also needed to maintain tolerance (15). DC responses to chronic inflammation may be regulated by a number of mechanisms. Therefore, changes in DC phenotype directly affect pathogenic and tolerogenic T cell responses in autoimmunity.

Another source of innate signals is the microbiome, and alterations in the composition of gut flora have been linked to human autoimmune disease (106, 107). For example, in T1D, studies of microbial composition in at risk individuals found that seroconverted subjects had lower diversity prior to disease onset (108), and lower levels of bacteria that produce the regulatory metabolite butyrate compared to controls that did not develop autoantibodies (109). In mouse models, where it is possible to go beyond correlation and dissect mechanism, manipulations of microbial content can alter autoimmune pathogenesis. In a model of arthritis, the presence of segmented filamentous bacteria in the gut induces systemic immune alterations, increased Th17 responses, and was necessary for full arthritis development; these bacteria alter diabetes pathogenesis in NOD mice as well (110, 111). Commensal flora is important triggers in a relapsing–remitting EAE model, advancing both pathogenic T and B cell responses (112). In NOD mice, the effects of microbiota on disease progression are complex. Mice lacking Myd88 (that cannot respond to most TLR signals) do not get diabetes, but NOD.Myd88^−/−^ mice in germ-free conditions do develop diabetes that can be blocked by transfer of defined microbial communities (10). Gender and hormonal differences also alter gut microbial composition and alter diabetes risk (113, 114). Indeed, the mechanisms by which microbial communities influence specific DC populations are just beginning to be defined (115), but these changes could in turn affect autoimmune pathogenesis.

Paradoxically, innate immune stimulators such as TLR agonists can sometimes inhibit autoimmunity. In NOD mice, weekly injections of many different TLR ligands, including poly(I:C) (TLR3 agonist), LPS (TLR4 agonist), or P40 protein from *Klebsiella* (TLR2 agonist), can block diabetes development if started at weaning (116). Poly(I:C) can block disease if treatment is started as late as 10 weeks of age, soon before the mice begin to develop diabetes. The effect of eliminating TLR signaling varies depending on the receptor. TLR9 expression is needed for development of diabetes and EAE in mouse models (117, 118). Conversely, TLR4 deficiency accelerates diabetes development in NOD mice (119). Therefore, the contribution of TLR signals to autoimmunity is complex, and can either inhibit or exacerbate disease.

## Effects of DC Maturation Thresholds on Development of Autoimmunity

The balance between tolerance and chronic inflammation associated with autoimmune disease can shift with relatively minor changes in DC phenotype. And although DCs can be sufficient for inducing autoimmune disease in several models, often other APC populations will drive disease in the absence of DCs. Costimulatory proteins on T cells and DCs act on multiple levels to affect autoimmune phenotypes, often altering the effector T cell to regulatory T cell ratio. Because some of the same costimulatory signals are needed for stimulation of effector T cells and Treg, a small change in level can alter the balance between these two cell types. CD28 and CD40 have disparate effects on inducing these T cell fates in NOD mice, as a loss of CD28 can restore diabetes in CD40L-deficient mice and alters the number of Tregs in those mice (120).

Changes in DC phenotype due to an inflamed system may be at a lower level than those observed due to treatment with exogenous stimulants or during acute infection. However, these changes can have dramatic effects on disease progression. For example, the less than twofold increase in CD40 on CD8^+^ DCs in NOD mice likely helps cause a switch from tolerance to effector responses in autoreactive T cells (12). CD40 expression levels affecting T cell fate is also observed in T cell responses to CD40 heterozygous mice. In a *Leishmania* infection model, CD40^+/−^ DCs-induced regulatory T cells while CD40^+/+^ DCs-induced effector T cells, while exacerbating or dampening disease, respectively (121). Indeed, these effects can be observed during both Treg and Teff stimulation. Lower levels of CD40 on DCs were associated with higher numbers of Tregs and inhibition of diabetes in an infection-driven model of type 1 diabetes (122). Expression of higher CD40 levels on APCs induced a greater proportion of effector T cells (123).

These subtle differences in DC phenotype leading to large differences in T cell outcomes present the possibility of a threshold effect. As shown with the *Leishmania* model, altering CD40 levels without completely losing those signals or overexpressing the protein can cause a profound change in T cell responses and alter the systemic immune response. DCs play an important role in the maintenance of immune homeostasis, and disrupting their phenotype by a small amount could lead to severe downstream alterations in effector responses. Thus, whether by genetic means or by inflammation from secondary sources, altered DC costimulation may push T cell and subsequent B cell responses to be aberrantly reactive to self-proteins, starting off autoimmune pathogenesis.

## Alterations in DCs due to Chronic Inflammation: Comparison to Infection

The inflammation that occurs during autoimmune pathogenesis may have parallels with the T cell responses during chronic infection because long-lasting effector T cell responses can alter the inflammatory state of a host, both via pro- and anti-inflammatory mechanisms. The generation of antimicrobial T cells can produce responses that have inflammatory effects on host tissues, as well as regulatory mechanisms to minimize host tissue damage (124). Autoimmune diseases that have environmental etiologies may have infectious triggers, and secondary autoimmunity can be triggered via molecular mimicry or pathogen-induced inflammatory environment (125). Indeed, altering the inflammatory status of DCs alters T cell responses and autoimmune pathology [reviewed in Ref. (126)]. Increased inflammation observed during autoimmune pathogenesis has several similarities to the host response to infection, and emerging studies have demonstrated that DC subsets play distinct roles during infection as well as during autoimmunity. We hypothesize that cellular responses including DC-driven immunity associated with a type 1 IFN signature may be similar regardless if the context is autoimmunity or chronic viral infections. The counterregulatory pathways associated with chronic stimulation could lead to an impaired ability of DCs to maintain Treg homeostasis.

It has been shown in the context of infection that different DC subsets can produce T effector responses through distinct mechanisms. For example, CD8^+^ DCs induce Th1 responses via CD70, while CD11b^+^ DCs induce Th1 responses in an IL-12-dependent fashion (127). DC subsets can also induce disparate T cell responses during the same infection. In the lung during influenza infection, CD103^+^ DCs induce effector CD8^+^ T cells via CD24, while CD11b^+^ DCs have reduced levels of CD24 and induce memory CD8^+^ T cells (128). Targeting of antigen to DC subsets can strong immunity in a variety of inflammatory settings. Targeting to cDC1 has long-lasting effects on T cell help for B cells, increasing responses against microbial pathogens, although this effect has not been tested for autoantigenic determinants (129). Targeting of antigen to DCs has also been harnessed in mouse models in anti-cancer therapeutics that demonstrate the power of DCs to turn T cell responses against self-tissues to good use. Protective immune responses against both HER2/neu breast cancer (cDC1) and melanoma (cDC1 and cDC2) have been induced by DC-targeted therapies (130, 131).

The type 1 interferons that generate a genetic signature often observed during autoimmune pathogenesis prior to overt disease are produced via distinct mechanisms in DC subsets. In response to measles virus, pDCs produce type 1 interferons through TLR7 or TLR9-MyD88-dependent pathway, while CD8^+^ DCs can produce type 1 interferons through Rig-I and MDA5 or TLR3–TRIF (132). DCs responding to respiratory syncytial virus also produce distinct T cell profiles. While infected human BDCA-1^+^ DCs induce a Th1 response in T cells, BDCA-3^+^ DCs induce Th2 and Treg responses (133). These distinct responses to pathogens based on the DC subset responding appear similar to what we have observed in NOD mice, wherein cDC1 produce effector responses while cDC2 are tolerogenic. Therefore, the inflammatory environment that is present during autoimmunity or infection may influence the phenotype of different DC subsets in distinct manners, and thus produce surprisingly different T cell responses.

## Blocking DC Alterations to Maintain T Cell Tolerance

Several costimulatory pathways in DCs have been manipulated to alter disease by affecting activation and proliferation of both effector T cells and regulatory T cells [reviewed in Ref. (134)]. The NOD mouse has been utilized to study several different mechanisms of blocking costimulatory pathways, both genetically and pharmacologically (120, 135, 136). Some of these therapies have been translated for use in type 1 diabetes patients: treatment with CTLA-4-Ig was successful in delaying disease, but was not successful in reversing disease course, with continued loss of C-peptide, a byproduct of insulin processing that is a marker of endogenous insulin production (137, 138). In mice, the TNF-family receptors OX40 and OX40L can be manipulated to prevent diabetes, probably via changes in regulatory T cells (139, 140). Although promising results with blockade of CD28 and CD40 in mouse models of SLE ameliorate disease and reduce the severity of disease indicators such as class-switched B cells and autoreactive-IgG, little efficacy was observed when targeting CD28, CD40, and ICOS in SLE patients (141–143). Blocking inflammatory cytokines that can alter DC phenotype has also been examined. One case report and one small study suggest that TNF-α blockade in patients with recent onset T1D may preserve beta cell function (144, 145). The timing of immunotherapies is often critical for successfully treating autoimmune disease. Several therapies were successful in reversing diabetes in NOD mice only when used during specific time intervals, whether those were several weeks prior to or post disease diagnosis (146).

## Implications for Designing New Therapies to Treat Autoimmunity

Given the relative lack of success at treating autoimmune disease with therapies targeting a single disease mechanism, it seems that combination therapies including both an antigen-specific component and an alteration of the inflammatory environment will likely be necessary to ameliorate disease. The central role of DCs in connecting innate and adaptive immune responses makes them an attractive target for at least part of that therapeutic regimen. Therefore, incorporation of the knowledge of the developmental and inflammatory signals that affect DC phenotype is critical in producing desired therapeutic outcomes. Understanding the differences in responses of specific DC subsets to these internal and external influences in the context of specific autoimmune diseases could lead to a significant increase in the effectiveness of therapeutics. Therapies designed to target antigen to specific DC subsets able to induce tolerance in that specific context may be more successful than non-specific delivery of antigen to many different APCs with varying activation states and phenotypes. Likewise, better characterization of the status of costimulatory molecules on the surface of DC subsets will help to determine which DC subsets will optimally induce tolerance. Knowing the leverage points at which DCs will produce effector responses or suppress T cell responses (especially for known or suspected initiating antigens) is an important characteristic of which therapeutic strategies can take advantage in ameliorating autoimmune disease.

## Conflict of Interest Statement

The authors declare that the research was conducted in the absence of any commercial or financial relationships that could be construed as a potential conflict of interest.
